# GeneChip analysis of human embryonic stem cell differentiation into hemangioblasts: an *in silico *dissection of mixed phenotypes

**DOI:** 10.1186/gb-2007-8-11-r240

**Published:** 2007-11-13

**Authors:** Shi-Jiang Lu, Jennifer A Hipp, Qiang Feng, Jason D Hipp, Robert Lanza, Anthony Atala

**Affiliations:** 1 Advanced Cell Technology, Worcester, MA 01605, USA; 2 Institute of Regenerative Medicine, Wake Forest University School of Medicine, Winston-Salem, NC 27157, USA

## Abstract

**Background:**

Microarrays are being used to understand human embryonic stem cell (hESC) differentiation. Most differentiation protocols use a multi-stage approach that induces commitment along a particular lineage. Therefore, each stage represents a more mature and less heterogeneous phenotype. Thus, characterizing the heterogeneous progenitor populations upon differentiation are of increasing importance. Here we describe a novel method of data analysis using a recently developed differentiation protocol involving the formation of functional hemangioblasts from hESCs. Blast cells are multipotent and can differentiate into multiple lineages of hematopoeitic cells (erythroid, granulocyte and macrophage), endothelial and smooth muscle cells.

**Results:**

Large-scale transcriptional analysis was performed at distinct time points of hESC differentiation (undifferentiated hESCs, embryoid bodies, and blast cells, the last of which generates both hematopoietic and endothelial progenies). Identifying genes enriched in blast cells relative to hESCs revealed a genetic signature indicative of erythroblasts, suggesting that erythroblasts are the predominant cell type in the blast cell population. Because of the heterogeneity of blast cells, numerous comparisons were made to publicly available data sets *in silico*, some of which blast cells are capable of differentiating into, to assess and characterize the blast cell population. Biologically relevant comparisons masked particular genetic signatures within the heterogeneous population and identified genetic signatures indicating the presence of endothelia, cardiomyocytes, and hematopoietic lineages in the blast cell population.

**Conclusion:**

The significance of this microarray study is in its ability to assess and identify cellular populations within a heterogeneous population through biologically relevant *in silico *comparisons of publicly available data sets. In conclusion, multiple *in silico *comparisons were necessary to characterize tissue-specific genetic signatures within a heterogeneous hemangioblast population.

## Abstract 2

Transcriptional profiling of human embryonic stem cells differentiating into blast cells reveals that erythroblasts are the predominant cell type in the blast cell population. In silico comparisons with publicly available data sets revealed the presence of endothelia, cardiomyocytes and hematopoietic lineages.

## Background

The establishment of human embryonic stem cells (hESCs) raised the possibility of being able to treat/cure many human diseases that are nowadays untreatable. This therapeutic potential, however, largely relies on the efficient and controlled differentiation of hESCs towards a specific cell type and the generation of homogeneous cell populations. Many differentiation protocols utilize the formation of progenitors through a stepwise approach. Thus, characterizing and understanding mixed populations of progenitor stages will be of increasing importance in stem cell research.

hESCs have been shown to be able to differentiate into a variety of cell types, including hematopoietic precursors and endothelial cells, *in vitro *under various culture conditions [1-9]. Hemangioblasts are the precursors of both hematopoietic and endothelial cells [10]. The existence of hemangioblasts was first demonstrated using an *in vitro *differentiation system of mouse ESCs. Replating of embryonic bodies (EBs) of mouse ESCs resulted in the formation of blast colony forming cells (BL-CFCs), which possessed hemangioblastic characteristics: BL-CFCs generated both hematopoietic and endothelial cells upon transfer to appropriate conditions [11,12]. Cells with hemangioblastic characteristics have been reported in both mouse and human adult tissues [13-18]. In an hESC system, Wang *et al*. [3] found that a fraction of a percent (0.18%) of CD45^neg^FVP cells with hemangioblast-like properties in hESCs derived from EBs. Zambidis *et al*. [8] demonstrated the formation of multi-potential colonies from hEBs, although it is unclear whether these colonies can be expanded and/or whether they have any functional activity *in vivo*. Umeda *et al*. [19] also identified the presence of CD34+/KDR+ bipotential cells in non-human (*Cynomolgus*) ESCs. Kennedy *et al*. [20] recently reported the generation of BL-CFCs from hESCs. However, the rarity of the cells with hemangioblast properties both from adult tissues and from ESC systems precluded comprehensive analysis of gene expression and comparison with other populations.

We have recently developed a two-step strategy that can efficiently and reproducibly generate blast colonies (BCs), the human counterparts of BL-CFCs, from hESCs [21]. These BC cells expressed gene signatures characteristic of hemangioblasts, and could be differentiated into multiple hematopoietic cell lineages as well as endothelial cells. When the BC cells were injected into animals with spontaneous type II diabetes or ischemia/reperfusion injury of the retina, they homed to the site of injury and showed robust reparative function of the damaged vasculature. The cells also showed a similar regenerative capacity in NOD/SCID β2-/- mouse models of both myocardial infarction (50% reduction in mortality rate) and hind limb ischemia, with restoration of blood flow in the latter model to near normal levels, demonstrating the functional properties of hemangioblasts *in vivo *[21]. In contrast to previous studies, these cells could be readily obtained in large scale, which allowed us to perform comprehensive analysis of gene expression in these cells and compare this with other cell populations from which the BC cells originated.

Microarrays assess the total amount of RNA in a population and can be influenced by a predominating cell type. Variation in the homogeneity of the population can influence the number of genes identified as differentially expressed. Here, we show how comparisons to publicly available tissues *in silico *can identify differentially expressed genes representative of the various cell types within a heterogeneous population.

In the current study, we analyzed the global gene expression profiles with robust multi-chip average (RMA) normalization to provide a relative value of gene expression between two samples. The first analysis consisted of direct comparisons with ESCs and their derivates (EBs and BCs). Genes enriched in BCs relative to hESCs revealed a genetic signature indicative of erythroblasts, suggesting that erythroblasts are the predominant cell type in the BC population. The next analysis consisted of multiple but biologically meaningful *in silico *comparisons to publicly available data sets that identified other progenitor cell types within the BC population. The significance of this microarray study is in its ability to assess and identify heterogeneous cellular populations through biologically relevant *in silico *comparisons.

## Results

### Strategy

Microarrays assess the total amount of RNA in a population and can, therefore, be influenced by a predominating cell type. Variations in the homogeneity of the population can influence the number of genes identified as differentially expressed, especially if both populations are relatively homogeneous. Here, we show how comparisons to publicly available tissues *in silico *can identify differentially expressed genes representative of the various cell types within the heterogeneous population of BCs.

We describe our method of assessing heterogeneous samples in three levels of analysis. The first level consists of making direct comparisons within the ESCs and their differentiated derivatives (EBs and BCs). The advantage of this technique is that it provides a kinetic-like relationship of changes in gene expression upon differentiation. The second level of analysis consists of indirect comparisons to a baseline, or reference tissue. Breast epithelium was chosen as a reference tissue because it represents a genetically distinct cell type that BCs are not capable of differentiating into. ESCs, EBs, and BCs were compared to breast epithelial tissue and differentially expressed genes were compared and contrasted to each other. Because genes that are up-regulated in BCs when compared to breast epithelia could represent those that are under-expressed in breast tissue, we removed those that were also up-regulated in a genotypically similar but different cell type (hESCs), when compared to breast epithelia.

The third level of analysis consists of comparing BCs to tissues they are capable of differentiating into as a way to mask that cell type's 'genetic signature' and reveal signatures of the more minor cell types. Samples were chosen based on type of GeneChip and their public availability - leukocytes, and endothelial and stromal cells. These biologically relevant comparisons identified tissue specific genetic signatures that would have otherwise been missed in the level I and II analyses.

**Figure 1:**
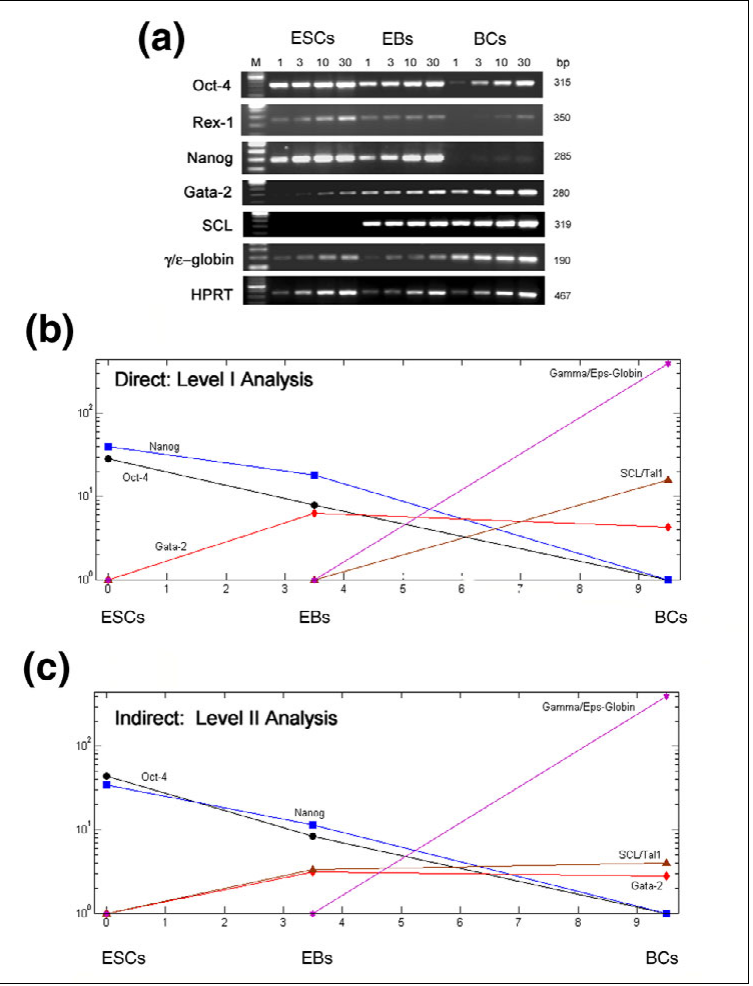
Validation of differentially expressed genes by RT-PCR in human ESCs, EBs and BCs. **(a) **Total RNA from human ESCs, EBs and BCs was used to construct cDNA pools, and the expression of genes was examined by semi-quantitative PCR. The number at the top of each lane indicates the amount (microliters) of cDNA used in the 50 μL PCR reaction. M = 100 bp DNA ladder. **(b) **Direct and **(c) **indirect analysis of differentially expressed genes matched the expression patterns obtained by RT-PCR. The fold change data are presented on the y-axis using logarithm-base-10.

The reliability of the microarray data generated from our multi-comparison analysis is demonstrated by the consistent identification of a set of genes among multiple comparisons, of which a subset of genes were confirmed by immunocytochemistry (Table 1) and RT-PCR (Figure 1). To summarize, comparing BCs to leukocytes identified genes involved in vasculogenesis, to endothelial cells identified genes involved in hematopoiesis, and to stromal cells identified genes involved in heart development.

**Table 1:** Characterization of hESCs and BCs by immunocytochemistry and Affymetrix arrays

		Level I	Level II	Level III		
				
	IC	BC direct	Epithelium	Leukocytes	Endothelium	Stromal
GATA1	+	+	+	+	+	+
GATA2	+	+	+	--	--	--
CD71	+	+	+	+	--	+
EPO-R	+	--	+	+	+	+
TPO-R	+	--	--	--	--	+
LMO2	+	+	+	+	--	+
β-Catenin	+	--	--	--	--	--
EGR1	+	--	--	--	--	--
Integrin α4	+/-	--	--	--	--	--
Integrin β1	+/-	--	--	--	--	--
VE-cadherin	--	--	--	+	--	--
E-cadherin	--	--	--	--	--	--
KDR	--	--	--	--	--	--
PECAM (CD31)	--	--	--	+	--	--
CD34	--	--	--	+	--	--
CD41	--	--	CD41b	--	CD41b	CD41b
CD43	--	--	--	+	+	+

The reliability of the microarray data generated from our multi-comparison analysis is demonstrated by the consistent identification of a set of genes among multiple comparisons, of which a subset of genes were confirmed by immunocytochemistry. For immunochemistry (IC): +, moderate to strong staining; -, negative staining; +/-, very weak staining. For Affymetrix arrays:+, detected as up-regulated in BCs; --, not detected as up-regulated in BCs.

### Level I analysis

#### Genes down-regulated upon differentiation of ESCs into EBs and BCs

We began our data analysis by verifying the expression of 'stemness' genes that are down-regulated in ESCs upon differentiation into EBs and BCs. We identified 87 genes that were down-regulated upon differentiation into EBs. Genes with the highest fold change include *SOX2, LEFTY1, GAL, NODAL, OCT4*, and *THY1*, which play a critical role in maintaining the undifferentiated status of ESCs [22-24]. To uncover enriched processes, data sets were analyzed by DAVID, a web-based tool that identifies over-represented biological themes in a data set based on their Gene Ontology (GO) terms. GO provides consistent descriptions of genes in terms of biological processes and molecular function. When these genes were clustered with DAVID based on their GO terms, processes involved in development, cell differentiation, and proliferation were identified. The genes identified in the development ontology were *DNA methyltransferase 3B*, *FGF2, THY1, SFRP2, LEFTY1, GREM1*, and *NODAL *(Additional data file 3).

We also identified 267 genes that were down-regulated upon differentiation of ESCs to BCs. These genes include *GAL*, *TDGF*, *NANOG*, *LEFTY1*, and *OCT4*, most of which are stemness genes [22-24]. When genes were clustered with DAVID using their GO terms, the processes included development, cell differentiation, and morphogenesis (Additional data file 4). These data demonstrate that *OCT4*, *NODAL*, *GAL*, and *THY1 *are initially down-regulated in stage 1 (ESCs→EBs) and are further down-regulated in stage 2 (EBs→BCs).

#### Genes up-regulated upon differentiation of ESCs into EBs

While the focus of this paper is to evaluate the pathways involved in hemangioblast differentiation, we begin by identifying those genes that were up-regulated in the early stage of differentiation into EBs (day 3.5) from which BCs were derived [21]. We identified 128 genes that were up-regulated upon differentiation of ESCs into EBs (Additional data file 5). These genes include *HAND1*, *WNT5*, *HEY1*, *LMO2*, *BMP4*, *TBX3*, and *MYL4*. Clustering these genes with EASE identified processes involved in development, transcription, organ development and system development, some of which are related to hemangioblastic differentiation. These genes include *SOX9, HOXB2, HOXB3, Neuregulin 1, LMO2 *[25] and *GATA2 *[26]. This data set also included numerous genes encoding transcription factors, such as *MESP1*, *HAND1*, *TBX3*, *GATA2*, *SOX7*, *SOX9*, *HOXB2*, and *HOXB3*.

#### Genes down-regulated upon differentiation of EBs into BCs

When EBs were compared to BCs, 185 genes were identified as down-regulated upon differentiation. This data set contained processes that were similar to those that were down-regulated upon ESC differentiation into EBs, such as tissue and organ development. The most significantly down-regulated genes included *NANOG*, *WNT5*, *OCT4*, *GAL*, *TDGF1*, *BMP4*, *endothelin receptor B*, and *VEFG *(Additional data file 6). These data demonstrate that *BMP4*, *WNT5*, and *HEY1 *are initially up-regulated upon differentiation into EBs but then down-regulated upon further differentiation into BCs.

#### Genes up-regulated upon differentiation of EBs into BCs

In contrast, 82 genes were up-regulated upon differentiation of EBs into BCs. The genes with the greatest fold change were hemoglobin genes and erythropoietic genes, such as *hemoglobins γ*, *ζ*, *α*, and *ε*, *Alas2*, *AFP*, *TUBB1*, *GYPA*, and *RHAG *(fold change, (FC) 31x-886x). Genes with moderate increases in expression (FC 6.2x-7.2x) *were KLF1, TAL1/SCL, GATA1 *and *CD71*. When genes were clustered with DAVID using their GO terms, processes characteristic of erythropoiesis (heme and porphyrin biosynthesis and oxygen transport) were identified (Additional data file 7).

#### Genes up-regulated upon differentiation of ESCs into BCs

There were 107 genes up-regulated upon differentiation of ESCs into BCs. Similar to the data set above (EBs→BCs), the genes with the greatest fold change (FC 29x-810x) were involved in hemoglobin synthesis (*hemoglobins γ*, *ε*, and *α*, *ALA2*, *GYPA*, and *TAL1/SCL*), similar to the comparison of EBs to BCs. In addition, this data set contained many key transcription factors involved in hemangioblastic differentiation, such as *GATA2 *[26], *LMO2 *[25] and *TAL1/SCL *[27,28] (Additional data file 8). *GATA2 *and *MYL4 *were up-regulated upon differentiation into EBs and remained at a constant level upon differentiation into BCs. EASE analysis of up-regulated genes in BCs identified biologically relevant themes, such as oxygen and gas transport, and development (*TAL1/SCL, KLF1, LMO2, GATA1*; Table 2).

**Table 2:** Gene ontologies for up-regulated processes in BCs versus ESCs

Gene category	List hits	EASE score
Oxygen transport (erythrocytic)	6	1.69E-09
Gas transport (erythrocytic)	6	1.69E-09
Transcription from Pol II promoter	7	1.64E-02
Regulation of transcription from Pol II promoter	5	2.38E-02
Development (developmental)	15	2.56E-02

EASE analysis of up-regulated genes in BCs identified biologically relevant themes, such as oxygen and gas transport, and development.

### Level II analysis

#### Genes enriched in ESCs

Since genes that are up-regulated in ESCs when compared to breast epithelia could represent those genes that are under-expressed in breast epithelium, we filtered out those that were also up-regulated in BCs when compared to breast epithelium. This analysis identified 2,108 genes, which comprised GO processes involved in cell cycle, DNA and RNA metabolism, and DNA replication, as expected (Additional data file 9). Genes with the highest fold change include *TDGF1*, *GAL*, *LEFTY1/2*, *OCT4*, and *NANOG *(FC 130.0x, 69.6x, 68.7x, 47.6x, 43.8x, and 31.5x). When this data set was clustered based on their GO terms, processes involved in development, cell differentiation and nervous system development were identified (data not shown). This data set was then analyzed with GenMapp, and then used for pathway analysis. Each genetic signature was assigned a color: ESCs, green; EBs, orange; and BCs, red. The ESC pathway confirms that most of the embryonic genes were not removed when compared to breast epithelial cells (Additional data file 1).

#### Genes enriched in EBs

Since genes up-regulated in EBs when compared to breast epithelia could similarly represent those that are under-expressed in breast epithelium, we also filtered out those that were also up-regulated in ESCs when compared to breast epithelium. We identified 939 genes as up-regulated in EBs relative to breast epithelium and filtered out those that are enriched in ESCs relative to breast epithelium (Additional data file 10). When these genes were clustered with DAVID, processes involved in development, transcription, wnt and frizzle signaling, cell cycle and blood vessel morphogenesis (*KDR, VEGF, Neuropilin-1 *and *2*, and *FLT1*) were identified The EBs also express genes involved in organ development, suggesting a heterogeneous mixture of cell types (*GATA2/4/5/6, BMP4, NCAM1, NOG, ISL2, NKX2.5*), and mesoderm genes (*HAND1, T-brachyury, MESP1*). Further examination of the data set identified multiple genes involved in the BMP signaling pathway in the differentiation of blood and endothelial cells. These genes include *BMPR1A*, *BMP4*, *T-brachyury*, *KDR*, *GATA2*, and *TAL1/SCL *[26,28].

#### Genes enriched in BCs

We identified 2,735 genes that were up-regulated in BCs relative to breast epithelium after removing genes that were enriched in ESCs when compared to breast epithelium and genes enriched in BCs when compared to breast epithelium (Additional data file 11). When genes were clustered based on their GO, we identified processes characteristic of lymphocytic cells (response to stimulus, defense response, immune response), erythrocytes (heme and porphyrin biosynthesis), coagulation, neurophysiology, development, and mesoderm and heart development (Table 3).

**Table 3:** Gene ontologies of up-regulated processes in BCs versus epithelial cells

Gene category	List hits	EASE score
Response to external stimulus (lymphocytic)	146	5.14E-09
Cellular process	490	4.74E-06
Response to biotic stimulus (lymphocytic)	94	4.87E-06
Ion transport	68	7.05E-06
Defense response (lymphocytic)	87	1.09E-05
Development (developmental)	165	5.05E-05
Hemostasis	19	1.15E-04
Blood coagulation (coagulation)	18	1.68E-04
Cell-cell signaling	60	2.23E-04
Response to abiotic stimulus (lymphocytic)	60	3.74E-04
Cyclic-nucleotide-mediated signaling	19	3.92E-04
Morphogenesis (developmental)	101	4.23E-04
Response to light	26	5.54E-04
Natural killer cell activation (lymphocytic)	6	7.08E-04
Second-messenger-mediated signaling	20	8.47E-04
Perception of light	25	9.29E-04
Synaptic transmission (neurophysiology)	31	9.63E-04
Cell communication	244	1.07E-03
Cation transport	46	1.46E-03
Transmission of nerve impulse (neurophysiology)	31	1.61E-03
Transport	159	1.65E-03
Response to radiation	26	1.73E-03
Organogenesis (developmental)	88	2.09E-03
Metal ion transport	36	2.15E-03
Perception of abiotic stimulus (lymphocytic)	35	2.34E-03
Vision	23	2.54E-03
Immune response (lymphocytic)	68	3.77E-03
Perception of external stimulus (lymphocytic)	37	4.60E-03
Cell surface receptor linked signal transduction	93	5.44E-03
Xenobiotic metabolism	10	6.19E-03
Sensory perception	34	6.34E-03
Heme biosynthesis (erythrocytic)	5	6.40E-03
Response to xenobiotic stimulus	10	8.00E-03
Skeletal development (development)	18	8.65E-03
Potassium ion transport	19	8.71E-03
G-protein signaling	15	9.32E-03
Pigment biosynthesis (erythrocytic)	6	1.10E-02
Monovalent inorganic cation transport	29	1.15E-02
Heme metabolism (erythrocytic)	5	1.16E-02
Negative regulation of natural killer cell activity (lymphocytic)	3	1.28E-02
Regulation of natural killer cell activity (lymphocytic)	3	1.28E-02
Anion transport	15	1.49E-02
Heart development (heart development)	6	1.63E-02
Histogenesis (developmental)	16	1.85E-02
Porphyrin biosynthesis (erythrocytic)	5	1.90E-02
Pigment metabolism (erythrocytic)	6	1.95E-02
Pigmentation (erythrocytic)	6	2.30E-02
Mesoderm development (mesoderm development)	7	2.37E-02
Cellular defense response (lymphocytic)	13	2.44E-02
Response to pest/pathogen/parasite (lymphocytic)	43	2.63E-02

When genes were clustered based on their GO, we identified processes characteristic of lymphocytic cells (response to stimulus, defense response, immune response), erythrocytes (heme and porphyrin biosynthesis), coagulation, neurophysiology, development, and mesoderm and heart development.

Genes that were up-regulated in BCs with respect to epithelia were characteristic of hematopoiesis (CD markers 5/6/9/38/41/48/55/71/74/84/244, *EPOR, GATA1/2/4/5, Tcr-α, natural cytotoxicity triggering receptor-1, 2 *and *3*), coagulation (*coagulation factor II, V, VII, XII, coagulation factor 2 receptor like 2,3/thrombin receptors, antithrombin 3, cyclooxygenase 1, plasminogen*), cardiac muscle (*NKX2.5, HAND1/2, GATA4, SOX6, TBX5*), smooth/skeletal muscle (*NOTCH1, smoothelin, acetylcholinesterase, desmin, SOX6*), synaptic markers (*cholinergic receptor, muscarinic 2 and 5, adrenergic α-1A-receptor, dopamine receptor D2, serotonin receptor 1B and 4, glutamate receptor Nmda 1 and 2A/B and C, gaba A receptor β1 and 2, purinergic receptor P2X 1 *and *2*), and hemangioblasts (*GATA2 *[26], *RUNX1 *[29], *LMO2 *[25] and *TAL1/SCL *[27,28]). Some of the genes identified in the coagulation ontology are not only involved in coagulation but also angiogenesis, such as *thrombin *[30], *plasminogen *[31], and possibly *coagulation factor 2 receptor like 2/3 *[30].

**Figure 2:**
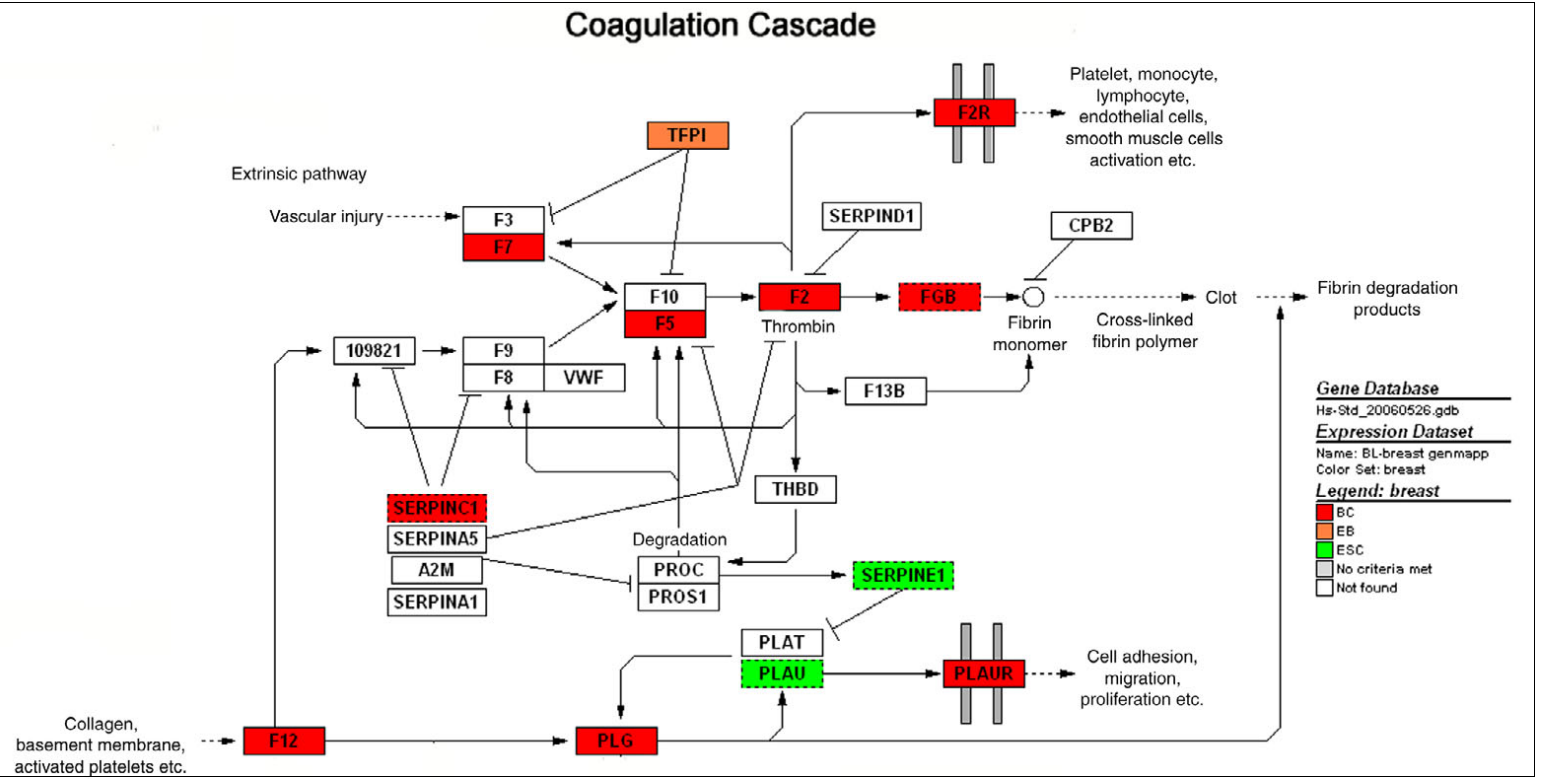
GenMAPP of complement and coagulation. Genes that are up-regulated in BCs (red), EBs (orange), and ESCs (green) compared to breast epithelia as baseline were mapped onto a pre-existing pathway. This pathway contains genes that are mostly up-regulated in BCs relative to breast epithelia.

**Figure 3:**
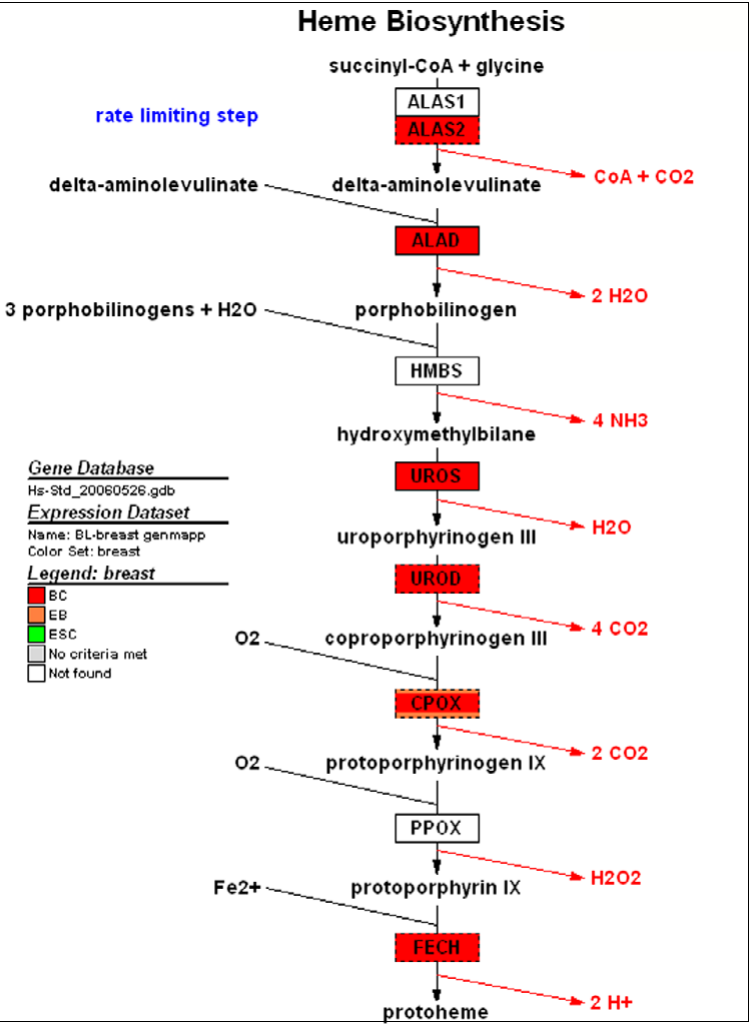
GenMAPP of heme biosynthesis: Genes that are up-regulated in BCs (red), EBs (orange), and ESCs (green) compared to breast epithelia as baseline were mapped onto a heme biosynthesis pathway. This pathway contains genes that are up-regulated in BCs relative to breast epithelia.

This was also recapitulated by DAVID analysis, which identified the following pathways as statistically over-represented: porphyrin metabolism, acute myocardial infarction, hematopoietic cell lineage pathways and calcium signaling pathways. GenMapp was then used for pathway analysis. Each genetic signature was assigned a color: ESC, green; EBs, orange; and BCs, red. GenMapp analysis of pathways involved in whole blood, bone marrow, coagulation and complement, heme and porphyrin synthesis are indicative of hematopoietic cell types (Figure 2). Genes identified in GenMapp's heme biosynthesis pathway are indicative of erythoblasts (Figure 3). We also identified myogenic (cardiac and smooth) pathways, which correlates with the GO analysis.

### Level III analysis

#### Genes enriched in BCs relative to leukocytes

We identified 2,101 genes that were up-regulated in BCs relative to leukocytes (after removing genes that were enriched in both hESCs and BCs when compared to leukocytes (Additional data file 12). When these genes were clustered based on their GO, we identified processes involved in development, nervous system development, blood vessel development and angiogenesis, and erythrocytes (Table 4). The presence of development ontology not only indicates a 'progenitor' status of BCs, but contains genes involved in hemangioblast development, such as *LMO2*, *TAL1/SCL*, and *RUNX1*. This comparison identified genes that are characteristic of endothelia (*PECAM1, VE-Cadherin, CD34, vWF, EPOR *[32], *endothelin 1 *[33], and *thrombin receptor*). There were also genes that indicate the presence of erythrocytes (*GATA1, spectrin *and *ankyrin*), blood vessel development (*neuropilin-1 *and *2, stabilin 1 *and *2*, *EGFR*, *FGF1 *and *6*, *NOTCH4*), and neurons/neuronal junctions (*glutamate receptor 1/6, serotonin receptor 1e/6, nestin, neurogenic differentiation 4, neuroligin 2, myelin basic protein, peripheral myelin protein 22*).

**Table 4:** Gene ontologies for up-regulated processes in BCs versus leukocytes

Gene category	List hits	EASE score
Development (developmental)	178	6.39E-09
Morphogenesis (developmental)	116	1.98E-08
Organogenesis (developmental)	105	5.04E-08
Cellular process	482	6.48E-08
Cell communication	257	4.78E-07
Cell adhesion	68	4.05E-06
Skeletal development (developmental)	25	4.44E-06
Cell-cell signaling	64	4.86E-06
Ion transport	62	8.10E-05
Circulation	20	9.47E-05
Metal ion transport	38	2.53E-04
Cartilage condensation	6	3.56E-04
Cation transport	46	5.97E-04
G-protein coupled receptor protein signaling pathway	61	8.03E-04
Regulation of blood pressure	9	9.06E-04
Neurogenesis (neuronal)	46	1.17E-03
Second-messenger-mediated signaling	19	1.32E-03
Cell surface receptor linked signal transduction	93	1.52E-03
Bone remodeling	9	2.01E-03
Ossification	9	2.01E-03
Cell homeostasis	15	2.07E-03
Cell ion homeostasis	14	2.27E-03
Ion homeostasis	14	2.27E-03
Transition metal ion transport	9	2.40E-03
Transport	151	2.97E-03
Homeostasis	15	3.10E-03
Transition metal ion homeostasis	7	3.37E-03
Receptor mediated endocytosis	7	4.20E-03
Metal ion homeostasis	11	4.48E-03
Homophilic cell adhesion	15	5.91E-03
Di-, tri-valent inorganic cation homeostasis	10	6.86E-03
Gas transport (erythrocytic)	5	7.51E-03
Oxygen transport (erythrocytic)	5	7.51E-03
Cation homeostasis	12	8.76E-03
Angiogenesis (blood vessel development)	8	1.10E-02
Cyclic-nucleotide-mediated signaling	15	1.13E-02
Transmission of nerve impulse (neurophysiology)	27	1.16E-02
Monovalent inorganic cation transport	28	1.19E-02
Di-, tri-valent inorganic cation transport	14	1.42E-02
Enzyme linked receptor protein signaling pathway	21	1.42E-02
Synaptic transmission (neurophysiology)	26	1.42E-02
Blood vessel development (blood vessel development)	8	1.44E-02
Iron ion homeostasis	5	1.64E-02
Blood coagulation (coagulation)	13	1.80E-02
Tyrosine kinase signaling pathway	15	1.99E-02
Signal transduction	177	2.18E-02
Potassium ion transport	17	2.47E-02
Hemostasis	13	2.77E-02
Pregnancy	8	2.89E-02
Axon guidance (neurophysiology)	6	4.00E-02

When these genes were clustered based on their GO, we identified processes involved in development, nervous system development, blood vessel development and angiogenesis, and erythrocytes.

Of particular note is the absence of leukocytic processes, such as response to stimulus and defense response identified in the level two analyses. Thus, this comparison allowed for the masking of the 'lymphocytic' signature and, thus, the identification of other endothelial and blood vessel development genes (*vWF*, *bradykinin receptor b1*, and *thrombin receptor*). When this data set was analyzed using GenMapp, more endothelial genes were mapped to the coagulation cascade pathway (vWF, bradykinin receptor b1, thrombin receptor-pathway; data not shown).

#### Genes enriched in BCs relative to endothelial cells

We identified 904 genes that were up-regulated in BCs relative to prostate-derived endothelium after filtering out those genes that are enriched in ESCs relative to endothelium (Additional data file 13). Comparing BCs to endothelial cells identified fewer genes when compared to other comparisons of BCs in level II and III analyses, and, thus, identified fewer GO terms. However, it did identify more erythrocytic processes (nine in total; Table 5) than the other comparisons. Another predominant theme in this data set was development (Table 5). By comparing BCs to a more mature yet similar cell type (adult endothelium), we were able to mask the endothelial signature, thus identifying predominantly development genes (indicating stem/progenitor type signature), such as *caudal type homeobox transcription factor 2 (CDX2), delta-like homolog (DLK1), lamin A/C, secreted frizzled-related protein 5 (SFRP5), patched (PTCH), dishevelled 2 (DVL2)*, and *even-skipped homeobox homolog 1 *(*EVX1*).

**Table 5:** Gene ontologies for up-regulated processes in BCs versus endothelial cells

Gene category	List hits	EASE score
Development (developmental)	84	2.10E-05
Oxygen transport (erythrocytic)	6	2.14E-05
Gas transport (erythrocytic)	6	2.14E-05
Heme biosynthesis (erythrocytic)	5	2.88E-04
Heme metabolism (erythrocytic)	5	5.56E-04
Morphogenesis (developmental)	51	6.08E-04
Organogenesis (developmental)	46	9.55E-04
Porphyrin biosynthesis (erythrocytic)	5	9.66E-04
Porphyrin metabolism (erythrocytic)	5	1.55E-03
Transport	77	1.89E-03
Pigment biosynthesis (erythrocytic)	5	2.83E-03
Pigment metabolism (erythrocytic)	5	4.70E-03
Pigmentation (erythrocytic)	5	5.46E-03
Hemostasis	9	9.09E-03
Learning and/or memory	4	1.67E-02
Skeletal development (developmental)	10	1.69E-02
Response to chemical substance	14	1.87E-02
Blood coagulation (coagulation)	8	2.05E-02
Hearing	7	2.16E-02
Perception of sound	7	2.29E-02
Synaptic transmission (neurophysiology)	14	2.69E-02
Ion transport	26	3.17E-02
Cell-cell signaling	25	3.20E-02
Cellular process	204	3.21E-02
Transmission of nerve impulse (neurophysiology)	14	3.36E-02
Response to abiotic stimulus (lymphocytic)	25	3.88E-02
Cartilage condensation	3	3.89E-02
Circulation	8	4.64E-02
Coenzyme and prosthetic group metabolism	9	4.98E-02

This comparison did not identify as many genes because of their similar origin and thus fewer gene ontologies. However, it did identify more erythrocytic processes than the other comparisons.

#### Genes enriched in BCs relative to stromal cells

The BCs were then compared to prostate-derived stromal fibromuscular (CD49a immunoselected) tissue. This comparison identified the most number of genes (3,277 genes; Additional data file 14), and had the most diverse GO terms (lymphocytic, developmental, erythrocytic, coagulation, synapses and neurogenesis, and heart development; Table 6). This data set contained lymphocytic processes according to their GO (response to stimulus, defense response) and numerous lymphocytic markers (CD 6/38/41/43/48/55/61/71/84/244, *immunoglobulin genes heavy constant γ1, constant κ, constant λ1*, and *CD 158A/B/D/F/H (killer cell immunoglobulin-like receptor*)). This comparison identified genes involved in hemangioblast differentiation (*TAL1/SCL, LMO2, RUNX1*), endothelial genes (*neuropilin 1 *and *2*), and coagulation genes (*fibrinogen α and β chain, coagulation factor 5, plasminogen*, but not *KDR*, *FLT1*, *CD4*, *PECAM*, *VE-Cadherin*, *vWF*). Although EASE analysis for both epithelial and stromal comparisons identified similar heart GO terms, the stromal comparison identified different heart development genes, such as *MEF2C*, *aortic preferentially expressed (APEG1)*, *POU6F1*, *TBX1*, and *ryanodine receptor 2 *(cardiac).

**Table 6:** Gene ontologies for up-regulated processes in BCs versus stromal cells

Gene category	List hits	EASE score
Cell communication	328	7.47E-07
Cellular process	617	1.25E-06
Cell-cell signaling	79	4.71E-06
Response to external stimulus (lymphocytic)	160	1.38E-05
Ion transport	74	4.07E-04
Response to abiotic stimulus (lymphocytic)	72	4.61E-04
Transport	199	8.97E-04
Cation transport	56	1.08E-03
Defense response (lymphocytic)	95	1.09E-03
Metal ion transport	44	1.30E-03
Response to biotic stimulus (lymphocytic)	101	1.35E-03
Development (developmental)	192	1.55E-03
Intracellular receptor-mediated signaling pathway	8	1.72E-03
Organogenesis (developmental)	108	1.98E-03
Cell adhesion	72	2.17E-03
Morphogenesis (developmental)	118	2.98E-03
Synaptic transmission (neurophysiology)	35	3.04E-03
Signal transduction	238	4.29E-03
Transmission of nerve impulse (neurophysiology)	35	5.07E-03
Regulation of blood pressure	9	5.42E-03
Monovalent inorganic cation transport	36	6.29E-03
Steroid hormone receptor signaling pathway	7	6.65E-03
Homeostasis	17	6.77E-03
Cell surface receptor linked signal transduction	113	8.56E-03
Perception of abiotic stimulus (lymphocytic)	39	1.09E-02
Hemostasis	17	1.10E-02
Second-messenger-mediated signaling	20	1.18E-02
Potassium ion transport	22	1.19E-02
Cyclic-nucleotide-mediated signaling	18	1.24E-02
Blood coagulation (coagulation)	16	1.39E-02
Response to chemical substance (lymphocytic)	31	1.45E-02
Heme biosynthesis (erythrocytic)	5	1.48E-02
Gas transport (erythrocytic)	5	2.00E-02
Oxygen transport (erythrocytic)	5	2.00E-02
Neurogenesis (neuronal)	51	2.02E-02
Cell homeostasis	15	2.31E-02
Perception of external stimulus (lymphocytic)	41	2.41E-02
Estrogen receptor signaling pathway	4	2.48E-02
Heme metabolism (erythrocytic)	5	2.61E-02
Homophilic cell adhesion	16	2.74E-02
Pigment biosynthesis (erythrocytic)	6	2.84E-02
Circulation (erythrocytic)	17	2.94E-02
Olfaction	9	3.02E-02
Response to pest/pathogen/parasite (lymphocytic)	52	3.19E-02
Acute-phase response (coagulation)	6	3.43E-02
Skeletal development	19	3.72E-02
Sensory perception	37	3.86E-02
Disaccharide metabolism	3	3.86E-02
Enzyme linked receptor protein signaling pathway	24	3.96E-02
Heart development (heart development)	6	4.09E-02

This comparison identified the most number of genes and had the most diverse gene ontologies.

When these genes were clustered for pathways analysis with DAVID, we identified Nfat, hypertrophy of the heart and Alk in cardiac myocytes as a statistically over-represented pathway (data not shown). GenMapp identified a similar pathway involved in myometrial contraction and calcium regulation in the cardiac cell (data not shown). These genetic signatures from this analysis would indicate the presence of progenitors (indicated by the number of developmental genes) of erythrocytes, leukocytes, neurons/neuronal-muscular junctions, and cardiomyocytes. Thus, comparing BCs to stromal cells masked a connective tissue-like signature, allowing for the identification of tissue-specific processes.

#### Ingenuity analysis

**Figure 4:**
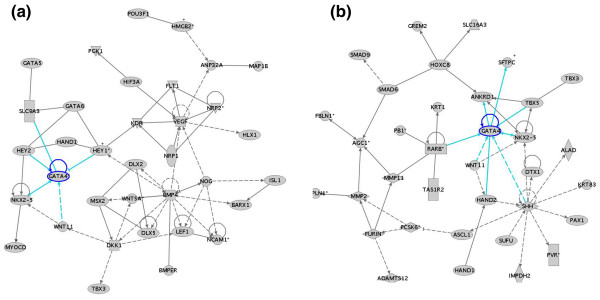
Ingenuity pathway analysis shows a network of genes expressed in EB and BC data sets from the level II analysis. This network contains nodes (genes/gene products) and edges (relationships between the nodes). The shaded genes, known as focus genes, were identified by microarrays and are the starting point to generate the network. The asterisks indicate that duplicates were identified in each dataset. The EB data set contains a network of genes (VEGF, GATA4, BMP4) that are interconnected and involved in blood vessel development (VEGF), heart development (GATA4), and cellular development (BMP4). The BC data set identified a network of genes involved in cardiovascular development (SHH, RARB, TBX5, WNT11) acting through GATA4. The intensity of the node color indicates the degree of expression. Nodes are displayed using various shapes that represent the functional class of the gene product (diamond-enzymes, ovals-transcription factors, triangles-kinase, circles-others). A solid line indicates a direct interaction while a dashed line indicates an indirect interaction. A line without an arrowhead indicates binding and a plus sign indicates that othernetworks contain this gene product.

**Figure 5:**
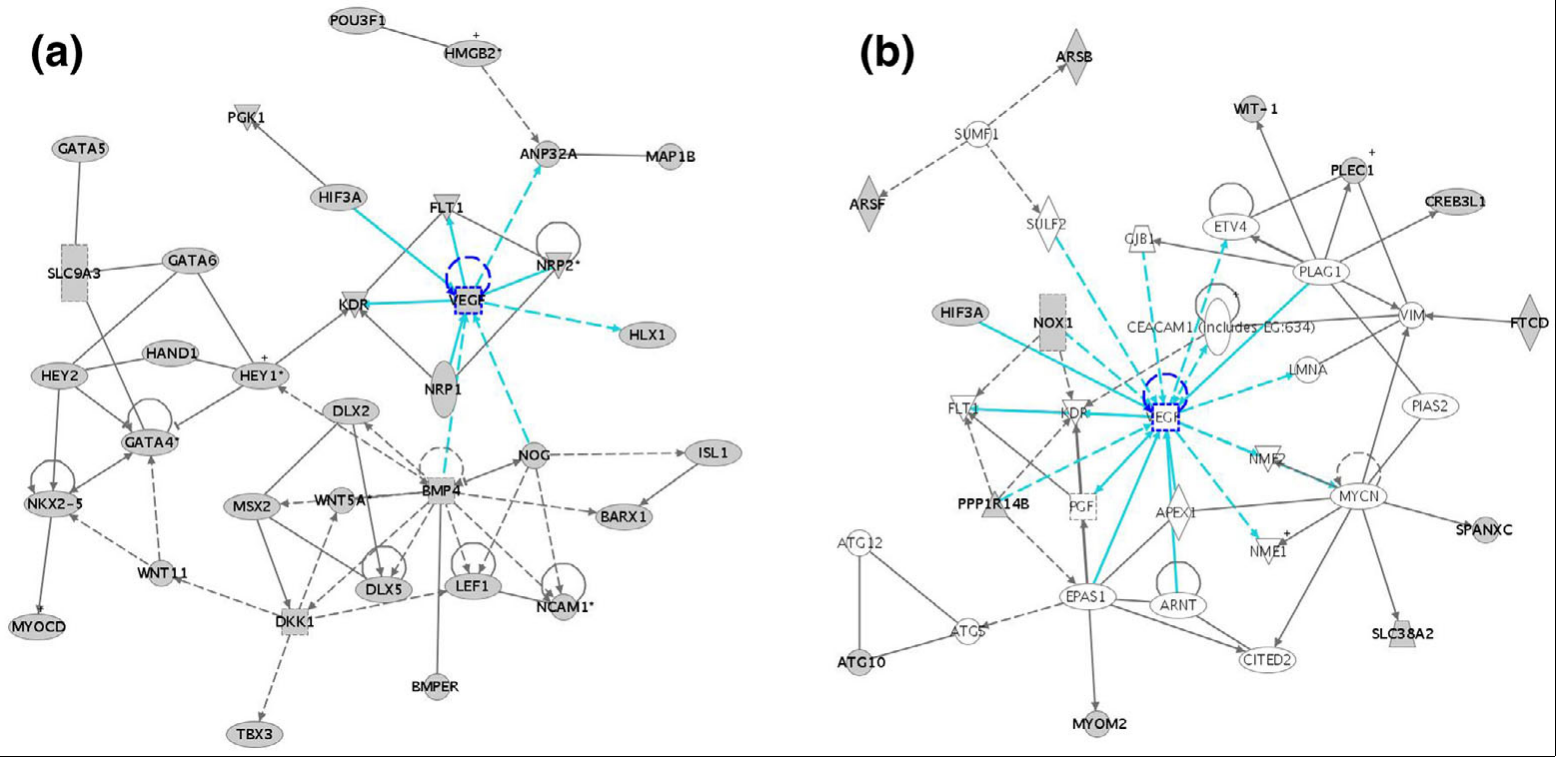
Ingenuity pathway analysis identified a network of genes expressed in EB and BC data sets associated with VEGF. In the EB data set, VEGF is associated with 35 'focus' genes, including KDR, FLT1, NRP1, and NRP2. In the BC data set, 13 focus genes were associated with VEGF, even though it is not present in the data set. The intensity of the node color indicates the degree of expression. Nodes are displayed using various shapes that represent the functional class of the gene product (diamond-enzymes, ovals-transcription factors, triangles-kinase, circles-others). A solid line indicates a direct interaction while a dashed line indicates an indirect interaction. A line without an arrowhead indicates binding and a plus sign indicates that othernetworks contain this gene product.

To identify signaling pathways involved in hemangioblast differentiation, each of the data sets was analyzed by Ingenuity. Ingenuity is a program that converts large data sets into networks containing direct and indirect relationships between genes based on known interactions in the literature. Genetic networks were created using the EB and BC data sets. The EB data set from the level 2 analysis contained a network of genes (*VEGF, GATA4, BMP4*) that are interconnected and involved in blood vessel development (*VEGF*), heart development (*GATA4*), and cellular development (*BMP4*) (Figure 4a). For example, *Bmp-4 *has been shown to promote blood vessel development by increasing VEGF production [34] and VEGF induces or binds to KDR FLT1, NRP1, and NRP2 [35-38]. This network suggests that BMP4 inhibits cardiac development by increasing HEY1, a transcriptional repressor of GATA4 and 6 (Figure 5a) [39,40], and inhibits heart development by inducing DKK1 (dickkopf homolog 1) [41], which then inhibits *WNT11 *mRNA expression, GATA4, and NKX2-5 [42-44]. In conclusion, this network suggests that BMP4 induces blood vessel development through VEGF signaling and inhibits cardiac differentiation through HEY1 and DKK1.

We then looked for these and other signaling pathways in the level 2 BC data set. Here we identified genes involved in cardiovascular development (*SHH *[45], *RAR-B, TBX5 *[46], *WNT11 *[43]) acting through *GATA4 *(Figure 4b). However, unlike the EB data set, we did not identify the cardiac repressor *HEY1*, *VEGF *and *BMP4 *in the BC data set. Instead, the BC network contained cardiac and skeletal genes, such as *HAND2 *and *ANKRD1 *[47-50], and *HIF3A*, an inhibitor of VEGF expression [51] (Figure 5b). Thus, these networks demonstrate that when EBs differentiate into BCs, we see some angiogenic and some cardiac pathways.

**Figure 6:**
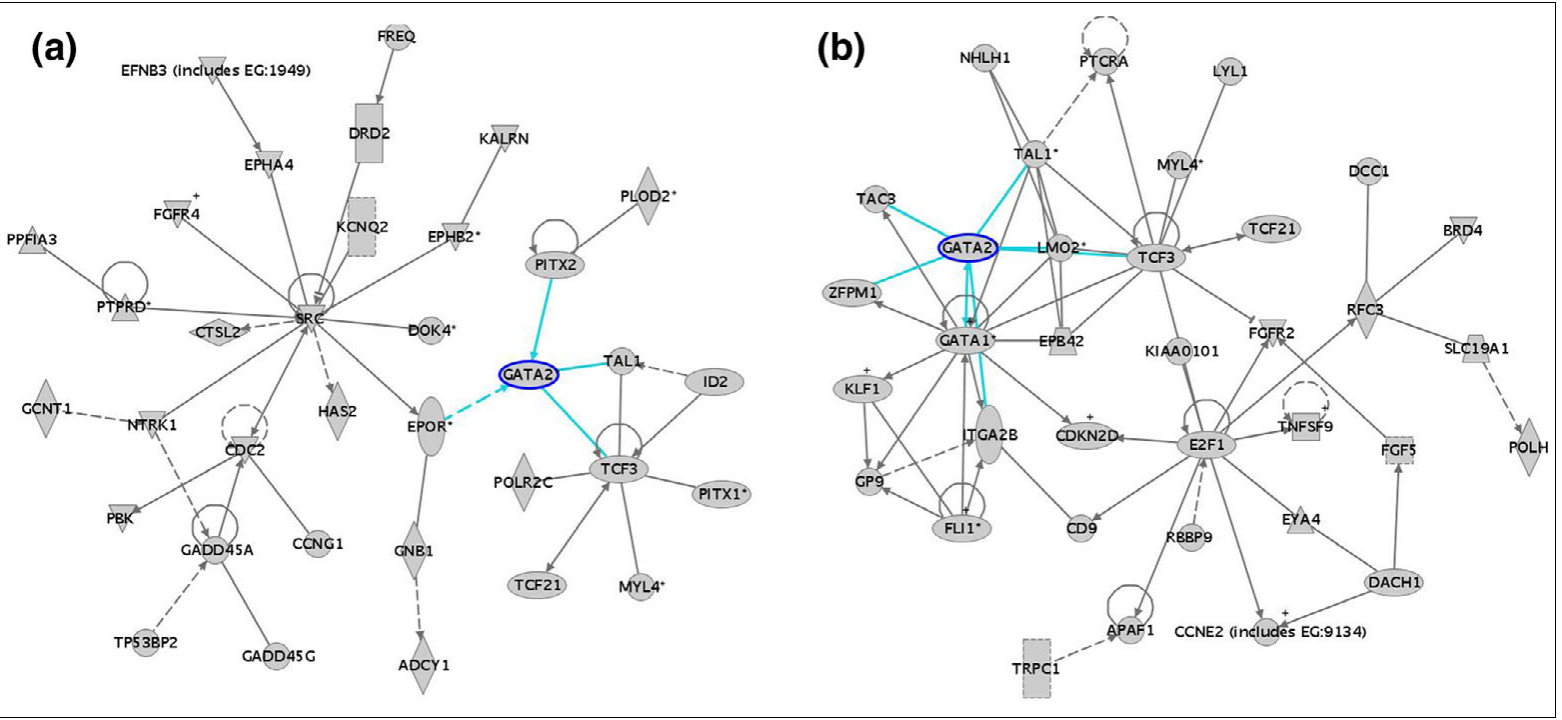
Ingenuity pathway analysis of a network of genes expressed in EB and BC data sets associated with GATA2. In the EB data set, the GATA2 network contained EPOR, TAL1, TCF3 and PITX2. In the BC data set, the GATA2 network contained the hemangioblastic and hematopoeitic genes TAL1, LMO2, FOG/ZFPM1, IGTA2B/CD41/GPIIb, and GATA1. The intensity of the node color indicates the degree of expression. Nodes are displayed using various shapes that represent the functional class of the gene product (diamond-enzymes, ovals-transcription factors, triangles-kinase, circles-others). A solid line indicates a direct interaction while a dashed line indicates an indirect interaction. A line without an arrowhead indicates binding and a plus sign indicates that othernetworks contain this gene product.

Another signaling network we identified as differentially expressed between EBs and BCs is the network containing *GATA2*. GATA2 has been shown to play a vital role in hemangioblast development [26] by up-regulating BMP4, KDR, and TAL1/SCL expression. In the EB data set, the GATA-2 network contained *EPOR, TAL1/SCL, TCF3 *and *PITX2 *(Figure 6a). Pitx2 is a homeobox gene involved in regulating the balance between proliferation and differentiation of progenitor cells [52] and is highly expressed in EBs (FC 24x) and is absent in BCs. PITX2 is not only rapidly down-regulated upon hematopoietic stem cell differentiation [52], but may also promote hemangioblast differentiation by inducing GATA2 expression [53]. In the BC data set, the *GATA2 *network contained the hemangioblastic and hematopoeitic genes *TAL1/SCL *and *LMO2 *[25,27,54], *FOG/Zfpm1*[55], *CD41/GPIIB/Igta2B *[56] and *GATA1 *[57] (Figure 6b).

A predominant network we identified in all three data sets of the level III analysis involved *GATA1*. GATA1 is a globin transcription factor and is present in all BC but not EB data sets. For example, we see *GATA1 *interacting with other nuclear genes, such as *TAL1/SCL*, *LMO2*, and *KLF1*, which induce erythropoetic genes such as the hemoglobin family (*HBG1*, *HBG2*, *HBE*, *HBB*, and *HBZ*), heme synthesis (*ALAS2*), and genes expressed on the cell surface of RBC (*Ankyrin 1, Rh blood group, glycophorin A, erythrocyte membrane protein band 4.2*) (Additional data file 2).

## Discussion

To delineate the mechanisms involved in the development of hemangioblasts from murine ES cells, Lugus *et al*. [26] performed global gene expression profiling of mouse Flk1+ cells that can form BL-CFCs. However, no such analysis has been reported for the human counterpart due to availability of such cells. In the present study, we carried out a large scale transcriptional l analysis to profile undifferentiated hESCs, early stage EBs, and BCs as an initial effort to understand the differentiation program of hESCs towards hemangioblasts. The availability of a well-annotated genome database for hESCs, EBs and BCs will provide a foundation that allows one to map and identify genes involved in human hemangioblast development, and to optimize conditions for efficient generation of hemangioblasts from hESCs. Our studies in general are consistent with previous reports that a cluster of genes (*OCT4, NANOG*) expressed at significantly high levels in hESCs were down-regulated in early stage EBs and BCs [23,58-63], whereas some genes restricted to endothelial and hematopoietic cells (neuropilins, *LMO2*, *GATA1 and 2*, *TAL1/SCL *and globins) were up-regulated dramatically in BCs and even in early stage EBs [2,23,26,64].

Our previous study has shown that BCs contain a mixed progenitor population of cells capable of forming hemangioblasts, and hematopoietic and endothelial cells [21]. To assess the heterogeneous populations in BCs, comparisons to publicly available data sets were performed *in silico*. Biologically relevant comparisons allowed us to mask particular genetic signatures within the heterogeneous population, allowing us to identify others, such as myogenic, vasculogenic, and hematopoietic progenitors within BCs. Our level I analysis compared hESCs to their differentiated progeny, providing a kinetic-like relationship of gene expression. When comparing the hESCs to BCs, many of the down-regulated genes were involved in development, cell differentiation, and morphogenesis. The predominant up-regulated genes identified in this level I analysis were those involved in the development of hemangioblasts and primitive erythroblasts. These genes include those encoding the transcription factors *TAL1/SCL, LMO2 *and *GATA1 *and *2*, heme synthesis genes, genes encoding erythroblast membrane proteins, and embryonic and fetal globin genes, but very low levels of adult globin gene, suggesting the yolk sac primitive status of BCs. This observation is consistent with findings obtained from both mouse and zebrafish models, in which hemangioblasts were first developed from primitive streaks and yolk sacs [65,66]. The results may also represent the fact that BC expansion medium contains several hematopoietic cytokines, which could push the developmental pathway towards the hematopoietic lineage, although BCs harvested at day 6 retain the potential of differentiating into endothelial cells under appropriate conditions [21]. Optimization of BC expansion conditions would, therefore, be valuable to keep these cells bipotential.

When applying this technique of multiple tissue type comparisons, some of the genes that are identified as 'up-regulated' in our tissue of interest could be 'under expressed' in the reference tissue. We controlled for this by filtering for those 'under expressed' genes with a comparison to a genotypically similar but different tissue type. For example, in our level II analysis, we identified those genes that are up-regulated in BCs relative to breast epithelial cells (3,700 genes) and then removed those genes that were up-regulated in hESCs relative to epithelial cells (2,735 genes). This removed 965 genes that could be thought of as being up-regulated in both BCs and hESCs relative to breast epithelium or as genes that are under-expressed in breast epithelium. GO analysis of this data set identified cell cycle genes as the predominant theme. This observation correlates with their biology because the BCs and hESCs are actively dividing cells *in vitro*, while the breast epithelium comprises relatively senescent cells freshly isolated from *in vivo*. Most importantly, we did not identify any tissue specific processes that we would expect to find in BCs.

GO analysis of the genes up-regulated in BCs with respect to epithelial cells after filtering out those that are up-regulated in hESCs relative to breast epithelial cells identified biological themes involved in erythropoiesis, as in the level I analysis (heme and porphyrin biosynthesis), but also the angiogenic components of coagulation, and synapses. This analysis identified genetic signatures representative of not just erythrocytes, as in the above level I analysis, but also the other cellular components of the BC population, such as muscle, cardiac, and hematopoietic cells and hemangioblasts. This *in silico *comparison to a biologically distinct reference tissue and filtering allows one to identify statistically significant genes that might otherwise be missed within a heterogeneous population.

In the level III analysis, biologically relevant comparisons were made *in silico *to adjust for particular cell types represented in the BC population. As in the level II analysis, this analysis also identified genetic signatures of the erythrocytic population in addition to other cellular components of BCs. When BCs were compared to leukocytes *in silico*, we identified a genetic signature representative of vasculogenesis, endothelia, neurons/synapses, hemangioblasts, and erythrocytes, with the relative absence of leukocyte genes. When BCs were compared to endothelia *in silico*, we identified a signature of erythrocytic and developmental genes with the relative absence of the vasculogenic signature. When BCs were compared to stromal cells *in *silico, we identified processes involved in hematopoiesis, synapses, angiogenesis/endothelia, development and more genes involved in cardiomyogenesis. Although EASE analysis for both epithelial and stromal comparisons identified similar heart GO terms, the stromal comparison identified different heart development genes. We believe that the epithelial comparison revealed more of the mesodermal aspect of BCs while the stromal comparison masked the mesodermal components and revealed more cardiomyocytic genes.

It has been shown that murine BL-CFCs are able to differentiate into hematopoietic, endothelial and smooth muscle cells, but failed to give rise to cardiomyocytes [67]. Molecular analyses showed that BL-CFCs expressed genes indicative of the hematopoietic and endothelial lineages, but not cardiomyocytes [26]. Kattman *et al*. [68] recently identified a cardiovascular progenitor with the same phenotype as the BL-CFC progenitor, brachyury+ and Flk-1+ cells from day 4.25 EBs (one day later than the BL-CFC progenitor) derived from mouse ESCs. These studies strongly suggest that BL-CFCs and cardiomyocytes, at least in the mouse ESC system, are derived from two different progenitors. Two other studies demonstrate the existence of multipotential progenitors for cardiomyocytes and muscle cells, but with different surface markers [69,70]. In the present study, several genes restricted to cardiomyocytes or their progenitor were detected with relatively high levels of expression in BCs. This observation suggests that human BCs may posses the potential to differentiate into cardiomyocytes, which will need further investigation. Alternatively, the purified BCs from multiple colonies could contain dissimilar blast clones originated from different differentiation stages, some of which may have the potential to develop into cardiomyocytes, as demonstrated recently by three groups in the mouse ESC system [68-70]. Simultaneous isolation and characterization of functionally distinct colonies and analysis of gene expression in these colonies might serve to determine whether human BCs posses the potential to differentiate into hematopoietic, endothelial and cardiomyocyte lineages.

## Conclusion

The identification and characterization of cell types within a heterogeneous population will be of increasing importance in stem cell research since differentiation protocols require the formation of progenitors through a multi-stage approach. Our previous study has shown that BCs contain a mixed progenitor population of cells capable of forming hemangioblasts, and hematopoietic and endothelial cells [21]. To assess the heterogeneous populations in BCs, comparisons to publicly available data sets were performed *in silico*. Biologically relevant comparisons allowed us to mask particular genetic signatures within the heterogeneous population, allowing us to identify others, such as myogenic, vasculogenic, and hematopoietic progenitors, within BCs. The significance of this microarray study is in its ability to assess and identify cellular populations within a heterogeneous population through biologically relevant *in silico *comparisons of publicly available data sets. In conclusion, multiple *in silico *comparisons were necessary to characterize tissue-specific genetic signatures within a heterogeneous hemangioblast population.

## Materials and methods

### hESC culture and BC growth

Culture of hESCs and growth of BCs were as reported previously [21]. In brief, undifferentiated hESCs (H1, H9) were cultured with inactivated mouse embryonic fibroblast cells in complete hESC media until they reached 80% confluence. Undifferentiated hESCs were dissociated by 0.05% trypsin-0.53 mM EDTA (Invitrogen, Carlsbad, CA, USA) for 2-5 minutes and collected by centrifugation at 1,000 rpm for 5 minutes. To induce hemangioblast precursor (mesoderm) formation, hESCs (2-5 × 10^5 ^cells/ml) were plated on ultra-low dishes (Corning, Corning, NY, USA) in Stemline II media with the addition of BMP4 and VEGF_165 _(50 ng/ml; R&D Systems (Minneapolis, MN, USA)) and cultured in 5% CO_2_. Forty eight hours later, half the media was removed and fresh medium was added with the same final concentrations of BMP4 and VEGF, plus SCF, Tpo and FLT3 ligand (20 ng/ml; R&D Systems), and PTD-HoxB4 (1.5 μg/ml) to expand out BCs and their precursor. After 3.5 days, EBs were collected and dissociated by 0.05% trypsin-0.53 mM EDTA (Invitrogen) for 2-5 minutes, and a single cell suspension was prepared by passing through a 22G needle 3-5 times. To expand BCs, a single cell suspension derived from differentiation of 2-5 × 10^5 ^hESCs were mixed with 2 ml hemangioblast expansion medium plated on ultra-low dishes and incubated at 37°C in 5% CO_2 _for 6 days, and BCs were then collected and subjected to RNA isolation.

### Affymetrix GeneChip analysis

Total RNA was isolated from purified BCs, day 3.5 EBs and undifferentiated ESCs (from two hESC lines, H1 and H9) using the Qiagen (Valencia, CA, USA) RNAeasy kit and amplified as previously described [21]. A total of six microarrays were performed (two biological replicates per time point using different hESC lines). Fragmented antisense cRNA was used for hybridizing with human U133 Plus 2.0 arrays (Affymetrix, Inc. Santa Clara, CA, USA) at the Core Genomic Facility of University of Massachusetts. The validation of differentially expressed genes was confirmed by immunocytochemistry in our previous studies [21] and by semi-quantitative RT-PCR analyses (Figure 1). The data discussed in this publication have been deposited in NCBI's Gene Expression Omnibus (GEO) [71] and are accessible through GEO series accession numbers GSE8884 and GSE9196, in accordance with MIAME standards. The demographics of the publicly available GeneChip data sets are breast-derived epithelial cells (*n *= 7), leukocytes (*n *= 6), prostate-derived endothelial cells (*n *= 5), and prostate-derived stromal cells (*n *= 5). These samples were chosen based on their homogeneity (cells were immunoselected or enriched) and the number of replicates. These data sets were downloaded from the NCBI's GEO and are accessible through accession numbers GSE9086 (breast-derived epithelial cells), GSE9091 (leukocytes), GSE9090 (stromal), and GSE9089 (endothelia).

### Data analysis

Raw CEL files were provided by the Core Genome Facility of the University of Massachusetts and were then analyzed with a software package AffylmGUI (Affymetrix LIMMA, Linear Models for Microarray Data, Graphical User Interfaces) [72,73]. Within AffylmGUI, gene expression values were summarized with RMA. RMA adjusts for background noise, performs a quantile normalization, transforms the data into log base 2, and then summarizes the multiple probes into one intensity [74-76]. Quantification of relative differences in gene expression among the groups of interest was accomplished using AffylmGUI, the sister package of limmaGUI [72,73]. AffylmGUI reads the raw Affymetrix CEL files directly, summarizes the gene expression values using RMA, and then uses LIMMA to identify statistically significant differences in gene expression [77]. LIMMA fits a linear model for every gene (like ANOVA or multiple regression analysis), and adjusts *P *values for multiple testings [77]. Differentially expressed genes were identified with a B statistic >0. The B statistic, also known as a likelihood of odds (LOD) score is a moderated *t*-statistic with posterior residual standard deviations. Subsequent analyses were performed in Microsoft Excel and Microsoft Access.

### EASE

The application Expression Analysis Systematic Explorer (EASE) was used to determine biologically relevant themes in a list of differentially expressed genes. EASE identifies over-represented biological themes in terms of their GO [78]. GO was developed to provide consistent descriptions of genes in terms of biological processes and molecular function. Genes with a B value >0 are incorporated into EASE where each gene is matched to all possible GOs. The results of this analysis are compared to all possible GOs for all genes on the microarray platform and calculates a *P *value, based on a conservative variant of the Fischer's exact probability test. We selected those pathways/processes with *P *< 0.05.

### DAVID

DAVID (Database for Annotation, Visualization and Integrated Discovery) provides tools and statistical methods for uncovering enriched processes and pathways within diverse and disparate gene lists [79]. DAVID also identifies over-represented biological themes in terms of their GO [78] and provides tools to visualize the distribution of genes on BioCarta and KEGG pathway maps.

### GenMapp

GenMapp is designed to visualize gene expression data on maps representing biological pathways and grouping of genes. It consists of hundreds of pre-made pathway maps and is used to identify pathway level changes amongst multiple data sets [80]. In this program, data sets are assigned a color corresponding to cell type and the direction of gene expression changes.

### Ingenuity

Networks were constructed using Ingenuity Pathways Analysis (Ingenuity^® ^Systems, Redwood City, CA, USA). A data set containing gene identifiers of genes with a B > 0 was uploaded into the applications. These genes, called focus genes, were overlaid onto a global molecular network developed from information contained in the Ingenuity Pathway Knowledge Base. Networks of these focus genes were then algorithmically generated based on their connectivity. Each network is a graphical representation of the molecular relationships between genes/gene products. Genes or gene products are represented as nodes, and the biological relationship between two nodes is represented as an edge (line). All edges are supported by at least 1 reference from the literature stored in the Ingenuity Pathways Knowledge Base. The intensity of the node color indicates the degree of expression. Nodes are displayed using various shapes that represent the functional class of the gene product (diamond-enzymes, ovals-transcription factors, triangles-kinase, circles-others). A solid line indicates a direct interaction while a dashed line indicates an indirect interaction. A line without an arrowhead indicates binding and a plus sign indicates that othernetworks contain this gene product.

### RNA isolation and gene expression quantification by semi-quantitative PCR

Total RNA was isolated from hESCs, day 3 EBs and hemangioblasts (BCs) using an RNAeasy Mini Kit (Qiagen) following the procedure recommended by the supplier with DNase I digestion, which eliminates the contamination of genomic DNA. RNA was subjected to first-strand cDNA synthesis with SMART II and CDS primers (Clontech, Mountain View, CA, USA), using Superscript II reverse transcriptase (Invitrogen), and cDNA pools were constructed using the SMART cDNA synthesis kit (Clontech) as described previously [2,81]. Complementary DNA pools generated by the SMART procedure have been shown to preserve the relative abundance relationship of the original mRNA populations [82-84]. The DNA templates in cDNA pools of human ES cells, EBs and hemangioblasts were adjusted to equal amounts based on the relative expression level of the hypoxanthine phosphoribosyltransferase gene (HPRT) and gene expression quantification by semi-quantitative PCR was performed as described previously [2,81]. The sense and anti-sense primer sequences, and the corresponding cDNA PCR product sizes, are shown in Table 7. The conditions for PCR amplification were as described with annealing temperatures and concentrations of MgCl_2 _for each specific gene as shown below. PCR products (10 μl) were separated on 1.5 to 2.0% agarose gel and visualized by ethidium bromide staining. The relative expression levels in cDNA from hESCs, EBs and hemangioblasts were estimated visually.

**Table 7:** Primer sequences

Gene	Primer	Sequence	Annealing Tm (C)	MgCl_2 _conc. (μM)	Product size (bp)
HPRT	Sense Antisense	5'-CTTGCGACCTTGACCATCTTTGGA-3' 5'-GGCGTCGTGATTAGTGATGATGAACC-3'	58	1.5	467
GATA-2	Sense Antisense	5'-TATGTGCCGGCGGCTGCCCACGACTACA-3' 5'-GGCTCTTCTGGCGGCCGACAGTCTT-3'	60	1.5	280
SCL	Sense Antisense	5'-GAAGTGCTCCCCTCTGAAAGTT-3' 5'-GGCTATCTCTCCTCTGACCTCG-3'	58	1.5	319
β-Cluster globin genes	Sense Antisense	5'-GTYTACCCHTGGACCCAGA-3' 5'-GCAGCTTGTCACAGTGCAG-3'	56	2	190
OCT4	Sense Antisense	5'-GAAGGTATTCAGCCAAACGAC-3' 5'-GTTACAGAACCACACTCGGA-3'	55	2	315
Nanog	Sense Antisense	5'-TGCAAATGTCTTCTGCTGAGAT-3' 5'-GTTCAGGATGTTGGAGAGTTC-3'	55	2	285
Rex-1	Sense Antisense	5'-TGACAGGCAAGAAGCTTCCG-3' 5'-GCGTACGCAAATTAAAGTCCAGA-3'	55	2	350

### Direct and indirect comparison of microarray data of differentially expressed genes

The direct analysis (level I) consists of making comparisons between ESCs, EBs, and BCs. Since there are two possible comparisons for each cell type, for example, ESCs relative to EBs or ESCs relative to BCs, fold changes were determined by comparing each to a cell type that did not detect the gene in question. Fold change levels for *Oct-4 *and *Nanog *were determined by comparing ESCs to BCs and EBs to BCs. Fold change levels for *Gata-2 *were determined by comparing EBs to ESs and BCs to ESs. Fold change levels for *SCL/Tal1 *were determined by comparing BCs to ESCs. Fold change levels for γ/ε-globin were determined by comparing BCs to EBs. For the indirect (level II) analysis, fold changes were simply determined by comparing each cell type to breast epithelia. If more than one probe-set was identified as differentially expressed, fold changes were averaged.

## Abbreviations

BC, blast cell; BL-CFC, blast colony forming cell; DAVID, Database for Annotation, Visualization and Integrated Discovery; EASE, Expression Analysis Systematic Explorer; EB, embryoid bodie; ESC, embryonic stem cell; GEO, Gene Expression Omnibus; GO, gene ontology; h, human; RMA, robust multi-chip average.

## Authors' contributions

SJL and QF designed and performed the cell culture, differentiation and RNA isolation. JAH and JDH performed the microarray analysis. This study was conceived by JDH, SJL, and JAH. SJL and JAH wrote the manuscript with input from JDH, RL and AA.

## Additional data files

The following additional data are available with the online version of this paper. Additional data file 1 is a figure showing a GenMAPP generated embryonic stem cell pathway that corresponds to genes enriched in ESCs (green), EBs (orange) and BCs (red) with epithelial cells as a baseline. Additional data file 2 is a figure showing a predominant Ingenuity generated network that was identified in all four data sets of the level III analysis. *GATA1 *interacts with other nuclear genes such as *TAL1*, *LMO2*, and *KLF1*, which induceeythropoetic genes such as the hemoglobin family (*HBG1*, *HBG2*, *HBE*, *HBB*, *and HBZ*), and those involved in heme synthesis (*ALAS2*). Additional data files 3, 4, 5, 6, 7, 8, 9, 10, 11, 12, 13, 14 are tables listing genes that were up-regulated in ESCs, EBs, or BCs relative to each other or a specific reference tissue. Additional data file 3 contains a list of genes that are down-regulated upon differentiation of ESCs into EBs. Additional data file 4 contains a list of genes that are down-regulated upon differentiation of ESCs into BCs. Additional data file 5 contains a list of genes that are up-regulated upon differentiation of ESCs into EBs. Additional data file 6 contains a list of genes that are down-regulated upon differentiation of EBs into BCs. Additional data file 7 contains a list of genes that are up-regulated upon differentiation of EBs into BCs. Additional data file 8 contains a list of genes that are up-regulated upon differentiation of ESCs into BCs. Additional data file 9 contains a list of genes that are up-regulated in ESCs when compared to breast epithelia. Additional data file 10 contains a list of genes that are up-regulated in EBs when compared to breast epithelia. Additional data file 11 contains a list of genes that are up-regulated in BCs when compared to breast epithelia. Additional data file 12 contains a list of genes that are up-regulated in BCs when compared to leukocytes. Additional data file 13 contains a list of genes that are up-regulated in BCs when compared to endothelial cells. Additional data file 14 contains a list of genes that are up-regulated in BCs when compared to stromal cells. Additional data files 15, 16, 17, 18, 19, 20 are six CEL files of data from human U133 Plus 2.0 arrays (Affymetrix, Inc.) hybridized to RNA from purified BCs, day 3.5 EBs and undifferentiated ESCs (from two hESC lines, H1 and H9). Additional data file 15 contains a CEL file of undifferentiated ESCs, H1-GFP labeled. Additional data file 16 contains a CEL file of day3.5 EBs-H1 and additional datal file 17 contains a CEL file of BC derived from H1. Additional data file 18 contains a CEL file from undifferentiated ESCs, H9. Additional data file 19 contains a CEL file from day3.5 EBs-H9. Additional data file 20 contains a CEL file of BC derived from H9.

## Supplementary Material

Additional data file 1 GenMAPP generated embryonic stem cell pathway that corresponds to genes enriched in ESs (green), EBs (orange) and BCs (red) with epithelial cells as a baseline. Click here for file

Additional data file 2 *GATA1 *interacts with other nuclear genes such as *TAL1*, *LMO2*, and *KLF1*, which induceeythropoetic genes such as the hemoglobin family (*HBG1*, *HBG2*, *HBE*, *HBB*, and *HBZ*), and those involved in heme synthesis (*ALAS2*). Click here for file

Additional data file 3 Genes that are down-regulated upon differentiation of ESCs into EBs. Click here for file

Additional data file 4 Genes that are down-regulated upon differentiation of ESCs into BCs. Click here for file

Additional data file 5 Genes that are up-regulated upon differentiation of ESCs into EBs. Click here for file

Additional data file 6 Genes that are down-regulated upon differentiation of EBs into BCs. Click here for file

Additional data file 7 Genes that are up-regulated upon differentiation of EBs into BCs. Click here for file

Additional data file 8 Genes that are up-regulated upon differentiation of ESCs into BCs. Click here for file

Additional data file 9 Genes that are up-regulated in ESCs when compared to breast epithelia. Click here for file

Additional data file 10 Genes that are up-regulated in EBs when compared to breast epithelia. Click here for file

Additional data file 11 Genes that are up-regulated in BCs when compared to breast epithelia. Click here for file

Additional data file 12 Genes that are up-regulated in BCs when compared to leukocytes. Click here for file

Additional data file 13 Genes that are up-regulated in BCs when compared to endothelial cells. Click here for file

Additional data file 14 Genes that are up-regulated in BCs when compared to stromal cells. Click here for file

Additional data file 15 CEL file of undifferentiated ESCs, embryonic stem cell line H1-GFP, that were hybridized to human U133 Plus 2.0 arrays (Affymetrix, Inc.) Click here for file

Additional data file 16 CEL file of day 3.5 EBs, derived from H1, that were hybridized to human U133 Plus 2.0 arrays (Affymetrix, Inc.) Click here for file

Additional data file 17 CEL file of BCs, derived from H1, that were hybridized to human U133 Plus 2.0 arrays (Affymetrix, Inc.). Click here for file

Additional data file 18 CEL file of undifferentiated ESCs from embryonic stem cell line H9, that were hybridized to human U133 Plus 2.0 arrays (Affymetrix, Inc.). Click here for file

Additional data file 19 CEL file of day 3.5 EBs, derived from H9, that were hybridized to human U133 Plus 2.0 arrays (Affymetrix, Inc.). Click here for file

Additional data file 20 CEL file of BCs, derived from H1, that were hybridized to human U133 Plus 2.0 arrays (Affymetrix, Inc.). Click here for file
